# Bioethical Implications of Globalization: An International Consortium Project of the European Commission

**DOI:** 10.1371/journal.pmed.0030043

**Published:** 2006-01-24

**Authors:** Thomas E Novotny, Emilio Mordini, Ruth Chadwick, J. Martin Pedersen, Fabrizio Fabbri, Reidar Lie, Natapong Thanachaiboot, Elias Mossialos, Govin Permanand

## Abstract

The BIG project looks at some of the ethical concerns surrounding globalization and health.

The term “globalization” was popularized by Marshall McLuhan in *War and Peace in the Global Village*. In the book, McLuhan described how the global media shaped current events surrounding the Vietnam War [ 1] and also predicted how modern information and communication technologies would accelerate world progress through trade and knowledge development. Globalization now refers to a broad range of issues regarding the movement of goods and services through trade liberalization, and the movement of people through migration.

Much has also been written on the global effects of environmental degradation, population growth, and economic disparities. In addition, the pace of scientific development has accelerated, with both negative and positive implications for global health. Concerns for national health transcend borders, with a need for shared human security and an enhanced role for international cooperation and development [ 2]. These issues have significant bioethical implications, and thus a renewed academic focus on the ethical dimensions of public health is needed.

Future developments in science and health policy also require a firm grounding in bioethical principles. These core principles include beneficence; nonmaleficence (to do no harm); respect for persons and human dignity (autonomy); and attention to equity and social justice. According to the World Health Organization [ 3], global ethical approaches should (1) monitor and update ethical norms for research, as necessary; (2) anticipate ethical implications of advances in science and technology for health; (3) apply internationally accepted codes of ethics; (4) ensure that agreed standards guide future work on the human genome; and (5) ensure that quality in health systems and services is assessed and promoted.

## The Bioethical Implications of Globalization Project

The Bioethical Implications of Globalization (BIG) Project is a 42-month dialogue funded by the European Commission that involves a series of expert panel discussions on four specific globalization and health subject areas: (1) mobility of people; (2) technological globalization; (3) liberalization of trade; and (4) new global health threats (bioterrorism). In addition, BIG includes a multiple-round Delphi Process ( Box 1) to solicit input on these issues from a broad, interdisciplinary audience.

The project's purpose is both to raise short-term, practical considerations about globalization and health and to provide a longer-term, strategic perspective on the four selected public health–related issues. The final conclusions will be presented to a high-level meeting of European Union (EU) policy makers in 2006; these conclusions may then inform future research directions and stimulate additional critical thinking about globalization and its bioethical implications for health policy. This article presents preliminary results from the BIG Project.

## Mobility of People and Consequences for Health Systems

Mobility results from the increasing ease of domestic and international travel as well as from instantaneous access to information through the Internet and other electronic resources. Mobility may involve the pursuit of a better quality of life, development of markets for traded goods and services, return of resources to home countries, and improvement of professional and business networks.

However, migration may also affect psychological and physical health as a result of conflict, famine, poverty, and the insufficient cultural or economic integration of migrants within their new home society. It may contribute to the spread of infectious diseases across borders ( Figure 1). The recent epidemic of SARS was a classic example of an infectious disease propagated through the movement of people across borders; it required attention from the original site to control migration (quarantine) as well as vigilance by secondary sites to protect their populations ( Figure 2) [ 4]. For these and other reasons, the International Organization for Migration is increasingly concerned with migratory patterns and their health consequences in a globalizing world (for an illustration of the emerging conflict of ideas, see http://www.iom.int and http://www.noborder.org/iom/index.php).

**Figure 1:**
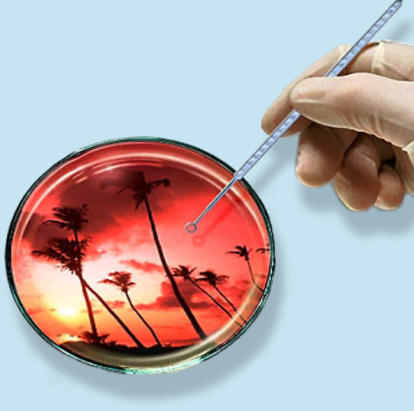
International Travel May Contribute to the Spread of Infectious Diseases across Borders

**Figure 2:**
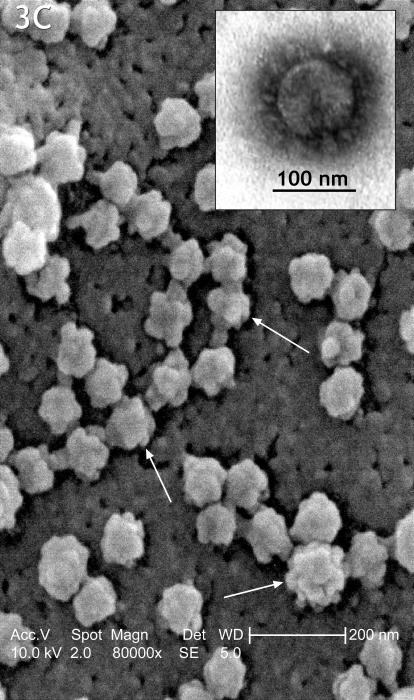
Scanning Electron Micrograph Showing the “Rosettelike” Appearance of the Matured SARS-CoV (Coronavirus) Particles (Arrows) This scanning electron micrograph emphasizes the form and structure of the virus particle, or virion, made visible with negative staining (inset) under transmission electron microscopy. Short and stubby spikes are visible on the virus surface. (Photo: CDC/Mary Ng Mah Lee, National University of Singapore, Singapore)

Cross-border health commerce is related to mobility. In Europe, this commerce is likely to increase as the EU enlarges to include Eastern and Central European nations. Such commerce may include the movement of health providers from East to West as well as “medical tourism” in pursuit of less costly or more accessible high-quality health care. In addition, international trade in illegal health products and inconsistent regulatory and safety standards for exports may threaten public health, especially in unregulated pharmaceutical markets.

Ethical concerns may also result from the vast growth in international tourist travel. Such travel now accounts for a twelfth of world trade, supporting an economy the size of a middle-income country [ 5]. Tourism may provide substantial economic benefits to many developing countries, and it may improve cultural understanding among travelers. However, these benefits require an ethical concern for the environment and for persons employed in the tourist industry.

The rights of nations to protect against infectious disease and unsafe medical practices, as well as the rights of human beings displaced by war, trafficking, and economic and cultural disruption, are critical concerns for health policy makers. Poverty and social disparities are key factors in the growth of global migration. Therefore, it is timely to consider whether mobility is a human right, and whether those who migrate have rights to health care in their new country. These questions should be considered by health policy makers within the ethical contexts of individual autonomy, social justice, nonmaleficence, and beneficence.

## Technological Globalization and Health: Information Technology and Genomics

Technology drives globalization and in turn is driven by globalization. However, there is considerable ambiguity as to the value of technological globalization, especially for health in low-income countries. The “digital divide” may be important in improving health or income disparities as the electronic revolution provides scientists and health workers in both the developed and developing world with unprecedented access to information. Much could be done to reduce information inequities for the developing world through collective international action, but new global governance mechanisms may be needed to achieve this goal for information technology [ 6].

Interestingly, the Internet is a structural necessity for financial and corporate globalization, but the same technology is used by nongovernmental organizations, political groups, and cultural movements to support grassroots social justice and human rights campaigns against these globalizing corporations. Neither side in this struggle would advocate limitations to the expansion of Internet technology, but both sides need to consider the bioethical implications of increased information access.

On the other hand, the ethical issues surrounding genomics (with both environmental and human concerns) are quite ambiguous. While there may be significant benefits to identifying genetically beneficial products or genetic determinants of disease, there are also concerns about altering natural environments and about collecting routine genetic information from general populations [ 7].

For example, some experts assert that genetically modified (GM) crops will significantly increase crop yields without requiring any additional farmland, thus preserving valuable rain forests and animal habitats. Other genetic innovations can reduce reliance on pesticides and herbicides that may contribute to environmental degradation. However, poor farmers may not be helped by GM technology. Five multinational companies (Monsanto [Creve Coeur, Missouri, United States], AstraZeneca [London, United Kingdom], DuPont [Wilmington, Delaware, United States], Pioneer Hi-Bred [Johnston, Iowa, United States], and Novartis [Basel, Switzerland]) control most of the GM seed market [ 8]. By linking their chemicals to seeds via GM technologies, these corporations extend markets for their fertilizers, herbicides, and pesticides. Farmers are not allowed to trade or save GM seed from one harvest to the next, and “terminator technology” (producing grains that are genetically modified so that they cannot be used to generate new crops) is under development. (See http://www.globalissues.org/EnvIssues/GEFood/Terminator.asp for more information on this technology.) Thus, ethical considerations of distributive justice and beneficence must be considered in the debate about the global applicability of GM crops.

For the pharmaceutical and health care industries, genetic testing could provide information about the shape of future markets and the possible tailoring of specific pharmacotherapy to genomic susceptibility. For governments, genetic testing may provide predictive information on a population basis that could aid future health care planning. Genetic information might also be similarly used by the insurance industry, but the identification of genetically “high-risk” individuals would likely interfere with their autonomy, in that they may not be able to purchase health insurance. For example, the Apolipoprotein E test may indicate that an individual has two copies of one form (allele e4) of the gene that leads to Alzheimer disease [ 9]. Could this information be used by insurance companies or possible employers to deny insurability, despite no current adverse health effects?

In the post-genomic era there is potential to both reduce and increase health inequities, but much will depend on how ethical issues are addressed. If interventions to increase the life span for those with access to high quality health care must compete with expensive investments in genetic research on infectious diseases (which affect the poor most of all), health inequalities may be amplified between those with access and those without access to health care. If research participants or patients in low-income countries have unequal access to information, they may not be properly informed about genetic testing and the counseling needed if adverse genetic information is found. Population-based genomic research may characterize groups of people in such a way that encourages discrimination. Such research may also lead to disputes about ownership of genetic resources from participant populations. Health professionals must have a solid grounding in bioethical issues as they make clinical decisions based on genetic information. However, health policy makers and global governance structures must also be accountable for the potential adverse consequences such decisions might engender.

One may ask: will genomic science really help developing nations? To what extent can benefits be shared? Will pharmaceutical and biotechnology companies invest in poor countries if they can make more money working on therapeutics for high-income countries? Thus, concern for the bioethical issues of social justice and beneficence arises. Genomics has the potential to be a global public good, but there is considerable uncertainty as to its bioethical justification in all cultures [ 10].

## Liberalization of Trade and its Effects on Health

In general, globalization helps liberalize trade through removal of import restrictions and tariffs, through removal of restrictions on trade in services, and through linkage of trade sanctions to the protection of intellectual property rights. All these activities may have an impact on population health.

Defenders of trade liberalization claim that this process is one of the most effective means of increasing a country's wealth and, by extension, population health. Even if this were always true, there may be specific policies that have particularly detrimental effects on health (such as opening markets to trade in manufactured tobacco products). Further, there may be an ethical argument based on social justice against some trade liberalization policies. If, for example, trade liberalization between rich and poor countries produces proportionally more wealth in rich countries compared with poor countries, this may suggest a socially unjust result of liberalization; poor countries' economies may not grow as fast as rich countries' economies in this situation.

The relationship between wealth and health is actually somewhat controversial: the so-called Preston curves demonstrate a dramatic relationship between health and economic prosperity up to about a Purchasing Power Parity of US$3,000 per capita per year [ 11]. However, there are cheap, cost-effective approaches to population health (such as vaccination, clean water, and sewage disposal) that may not be affected by the increase in Purchasing Power Parity. These approaches were relatively more important than economic development per se in early 20th-century interventions in developed countries, and they are likely to be more important for influencing health among developing country populations today than simple economic growth. On the other hand, high-intensity technological improvement rather than economic growth may be more important to health in rich countries compared with developing countries.

The concern for intellectual property rights in trade has been an extraordinarily contentious issue in recent years. Newer drugs that are effective against diseases in resource-poor but highly impacted countries, such as antiretroviral drugs against HIV, have been prohibitively expensive in these countries, in part because of patent protections. With the Trade-Related Intellectual Property agreement, patent protection became linked to trade policy; if countries in need of cheaper essential drugs did not conform to patent rules, trade retaliation from exporting countries might ensue. However, restrictions on poor countries' responses to legitimate public health emergencies may be unethical on the basis of distributive justice, nonmaleficence, and beneficence. Exceptions for public health emergencies (such as HIV/AIDS) under the Trade-Related Intellectual Property agreement include the right to compulsory licensing (local companies produce patented medicines in exchange for a royalty payment to the patent holder) or parallel importing (importing patented drugs sold more cheaply elsewhere) that will make essential medicines more available to highly impacted countries without fear of trade retaliation from the originating country [ 12].

The General Agreement on Trade in Services is a relatively new treaty that covers trade in health services [ 13]. The agreement has been severely criticized by some, who claim that it increases privatization of health care services and undermines public health care systems. However, given its ambiguities, the actual impact of the agreement on the health sector will be largely determined by the way in which the agreement is further specified in multinational commitments [ 14]. Social justice, equity, beneficence, and nonmaleficence will all come into play in the implementation of this treaty.

## New Global Health Threats, Focusing on Bioterrorism

Concerns for security against biological weapons have recently arisen among both poor and wealthy nations. Some, however, question the enormous sums now being spent to address the perceived threats due to bioterrorism even without strong evidence for actual threats. Even without such evidence, global bioethical principles at least suggest the need for a framework for consideration of distributive justice in this arena.

For example, should a nation with a limited supply of a vaccine against weaponized smallpox offer its stockpiles to a neighboring country that is under direct attack? This case is complicated by the fact that the infection could spread to its own territory. In the case of widespread biological attacks, which global governing agency, country, or other entity would be responsible for global resource allocation? Clearly, risks from bioweapons are trans-border, but resources may be unevenly and inequitably distributed, requiring a bioethically based policy determination on a global basis [ 15].

A further concern with respect to biomedical research is the issue of dual-use technology development for health benefits as well as for possible bioweapons. Governments must balance the secrecy necessary for security with the need for disclosure of information that is essential for research and development in health. It is very difficult to sequester new knowledge that might be applied to building biological weapons without simultaneously impeding research on defense against those bioweapons and on other beneficial biomedical advances. Most BIG Project scientists agree that the benefits of releasing scientific information in general outweigh the risk of its misuse. However, the scientific community needs to consider whether new codes of conduct are necessary or whether existing governance is sufficient to support a bioethical approach to research on possible dual-use technologies.

## Conclusion

Global bioethical challenges require careful theoretical deliberation and practical considerations for international health policies [ 16]. The BIG Project seeks to guide these processes in four selected areas of interest to the EU, so that the project results may be helpful to policy makers at local, national, and international levels.

The BIG Project has found that bioethical principles are important in considerations of migration, trade, information technology, genomics, and bioweapons threats. Globalization in these arenas is neither a right nor a wrong process, but it demands careful consideration of bioethical principles including social justice, beneficence, nonmaleficence, and individual autonomy. These concerns may not be immediately obvious to health policy makers, and thus the BIG Project results may help clarify the larger goals and purposes of bioethically based health policy development within the EU and elsewhere. More information about the BIG Project can be found at http://www.bigproject.org/project.htm.

**Box 1. The Delphi Method**

Delphi is a group communication technique designed to obtain opinions from a panel of selected experts on specific issues through the sending of questionnaires to be completed within a specified time. The experts are contacted individually and they do not know other group participants and their opinions—the aim is to submit the group participants to the same conditions. Participants do not meet personally, thereby avoiding undue influence. The process foresees the following points:

- Identification of the problem
- Selection of the relevant questions
- Preparation and delivery of the first questionnaire
- Analysis of the answers and elaboration of the first synthesis; preparation and delivery of a second questionnaire containing reformulated questions based on such elements as results from analysis of and comments upon the first questionnaire, illustrative of how other participants have answered previous questions
- Analysis and synthesis of answers
- Delivery of further questionnaires as required

The process is repeated a number of times, until a convergence of all group members is obtained. The process ends with analysis of the answers and formulation of the final report.

(Adapted from http://www.bigproject.org/dephi.htm)

## Abbreviations

BIG: Bioethical Implications of Globalization
GM: genetically modified
